# Indents

**Published:** 1913-03-15

**Authors:** 


### INDENTS.

☞Brief Articles of Technical or Scientific Value will receive notice and comment. Contributions invited.

*Are the Professions Overcrowded?* We are frequently asked by amateur photographers who contemplate joining professional ranks if it is true that the field is overcrowded. Our answer is that the profession is no different from others which attract a seemingly excessive proportion of the human race. At the rate law and medical schools turn out practitioners every year one does not wonder that the cry goes forth, How on earth are these legions to find anything to do when there's already an excess of lawyers and doctors? It is true that only a relative few attain success; this is not so much because the field is overcrowded as that those who fail to make good are either improperly equipped or are deficient in moral courage, resourcefulness and perseverance. The ambition to excel and to achieve great things, in spite of seroius obstacles, is not implanted in every man's breast. A powerful incentive, backed by genuine ability and sound business instinct, carries the day. To men of this caliber it does not matter if their line of business is overcrowded.—Editorial, *Photo-Era*, October, 1912.

*Method of Reenforcing Plaster Casts.* Many times we find that when our models are made they are very thin, too thin and weak to withstand the pressure necessary in closing the flask. We also find abutments breaking off the model in bridge work, etc. An easy, cheap and sure method to overcome this difficulty is to get some brass wire gauze, to be found at all tin shops. This gauze is that used for milk strainers, etc. For reenforcing crowns used as abutments in bridge work roll up a piece about one-half inch long and one-half the size of a lead pencil, and after dipping it into water gently work it into the crown when you are pouring the investment in to get the model. We will assume that you have a lower impression of a very thin ridge and that you are ready to make the model. Cut a piece of the gauze about two inches wide and long enough to go from one end of the model to the other. Then fold the gauze in the middle and as you pour the plaster into the impression work the smooth edge of the gauze (not the cut edge) into the plaster, holding it for a moment until the plaster begins to set.—J. M. Prime in *Brief.*

*Practical Suggestions.* Teach your patients to brush their gums as well as their teeth. In administering an anesthetic remember quietness is the secret of success.

In diagnosis, remember temperament should be observed, as each may require a different treatment.

In treating stubborn cases of abscess teeth, use a 50 per cent solution of sulphuric acid; if the abscess is hard to reach, use pressure. Protect the surrounding parts with cotton moistened with a soda solution, also wash out cavity after treatment with same solution.

Sandarac varnish used on aluminum plates before investing for vulcanizing will prevent discoloration.

In packing rubber for vulcanizing when a thick body is required, sprinkle in a little alloy as you pack the rubber, and the plate will not be porous. The general cause of porous plates is a leakage of steam during vulcanizing.—Gustav North in *Dental Brief.*

*Rosin Dissolved in Chloroform for Pulp Canal Filling.* Dr. J. R. Callahan, of Cincinnati, O., recommends first filling the cleansed and dehydrated pulp canal with a solution of one part rosin to three parts chloroform, pumping it thoroughly into the canal. Allow this to remain a few minutes and then remove the surplus with paper points, and complete the canal filling with a rosin solution to which sufficient pink gutta-percha has been added to make it of the consistency of chloro-percha as usually used. When using the rosin solution, in preparing the canal for filling, he uses chloroform instead of alcohol. He claims to be able by this method to not only perfectly fill the canal, but also the tubuli right up to the cementum. The rosin, he states, remains hard and insoluble in the body fluids, and makes a permanent and safe pulp canal filling. —*Dental Brief.*

*Wholemeal Bread.* The following eulogy on wholemeal bread occurs in the recently published reminiscences entitled "I Remember," by John Wm. Horsley, M. A., Oxon., Rector of St. Peter's, Walworth, Hon. Canon of Southwark, Mayor of Southwark.

"It was, I think, in 1872, when I came from Witney, where my fellow curate was the present Bishop of St. Albans, to have a day's fishing at Osney Mill, in Oxford. Lunching there with my friend the miller, biting because the fish did not bite, I remarked on the extreme goodness of the brown bread on his table. 'Yes,' he said, 'that is real bread, I have it made from the meal as it comes from the first pair of stones, and what most people prefer and buy is the residuum after all the flesh-forming ingredients have been taken away by subsequent processes. Pigs thrive on what ignorant people reject, and suffer by the rejection. Only,' he added with a smile, 'you must not let everyone know this, since by this craft we have our living! Much of our labor and expensive machinery would not be needed if people ate wholemeal bread.' Since then I have never eaten white bread when I could get anything else; and if you were at my table, though you would find both kinds provided, I should ask you whether you preferred 'bread or white-stuff.' The only time when I have had symptoms of indigestion or constipation has been when I have been in an hotel abroad and find only the lightest and whitest bread before me; and I have cured not a few in various ranks of life who complained of these ailments buy inducing them to discard white stuff. I mention this especially because some people talk of the emasculated flour being more digestible. The white carbon of the interior of the grain, which provides the heat-forming food and nothing else, has five envelopes, of which all but the exterior one give the nitrogenous or flesh-forming food, and only that outside skin or bran is indigestible. But it has an excellent mechanical effect in preventing constipation. As to general nutrition, I have always found that a few slices of wholemeal bread are sufficient for a meal, when with white stuff one seems to 'get no forrader' with a great number of slices. Of course, when one includes in one's dietary meat or other flesh-forming substances, the use of white stuff is not so starving; but, unfortunately, the poorest people, who can get the least meat, and have not learned to use lentils, haricots, or other flesh-forming foods, are just those prejudiced against any but the whitest bread, and this is one cause of general feebleness of their physique and possibly of their commonly bad teeth. When for ten years I was chairman of our Newington Workhouse, where we used twenty-six sacks of flour a week, baking for this and our other Poor Law institutions, I always longed to produce wholemeal bread, but knew well that a riot would have been the consequence. However, when for some thirteen years we fed all through the winter half of the year, in and from the crypt of St. Peter's, tens of thousands of the school children of the neighborhood, we never provided anything but wholemeal bread and wholemeal raisin bread, and it was only a new child that thought at first we were providing anything inferior. This had, I hope, an educative effect. Our London prisons were also, earlier than this, pioneers of bread reform, and if for any reason the doctor desired a prisoner to have white stuff, 8 oz. were given instead of the wholemeal 'sixer,' made in Coldbath Fields, and thence distributed. In private life I have known some—one friend a country rector, another a board school headmaster, another a masseuse—

who found they promoted both health and economy by having their own little steel hand-mill, buying a sack of corn and grinding the day's supply before breakfast. The rector told me that he saved the cost of the mill in the first sack of corn. This question of economy leads me to ask why wholemeal, in its many varieties, is no cheaper than the white stuff which has required more machines and more processes on the part of the miller? There may be a conclusive answer, and, if so, I should like to know it; but am convinced that even if the price of real bread were higher it would be more economical, as well as better, in its use."—*Dental Record.*

*Fused Inlays, Using Platinated Gold.* By the following process of platinizing shredded pure gold, its melting point may be raised so that pure gold may be melted into a mass of it without melting or changing its form.

Pour over any form of pure gold fibre, wool, moss, pellets or foil (a shredded pure gold is preferable) a 5 to 10 per cent. solution of platinum-chloride sufficient to dampen or wet all surfaces and pour or squeeze off the excess. The platinum chloride can be made by dissolving platinum in aquaregia (which is ¼ hydro-chloric and ¾ nitric acid mixed) and driving out the nitric acid by heat, redissolving in hydro-chloric acid and diluting with water. Next pour over it a solution of ammonium chloride and flow it through the mass a time or two to precipitate the platinum-chloride as platinum ammonium chloride, which forms a yellow precipitate over the surfaces of the gold. Take the water out of the mass by slowly heating or, preferably, by washing with alcohol. Then heat to a dull red, which changes the yellow platinum-ammonium chloride on the surfaces of the gold to amorphous spongy pure platinum, which combines with the gold when heated to a red heat, forming a gold and platinum compound.

The advantages of a shredded gold over a foil or pellets is that the former will not curl and warp away from the margins by the gold flowing on its surfaces, which the foil and pellets, when used in this way, will do. The platinized shredded gold draws the pure gold into its mass around all sides of each fibre, thus preventing the curling. The platinized gold is packed into the cavities in artificial stone models and bar gold fused into it. The advantages are that there is less error from shrinkage and that we have the flow that we have in pure gold fillings for finishing and more strength because of the added platinum, and all in half the time required for casting.—Dr. W. A. Price in *Dental Summary*.

*Causes of Mortality among Dentists, compared with other Professions.*

In the *Dental Record* for June of last year there was reproduced an interesting article by Dr. A. O. Ross, of Ohio, entitled "Causes of Mortality among Dentists, compared with other Professions." One of the author's findings was that disease of the heart was the most prolific cause of death, being about 20 per cent. of all causes, and greatly in excess of the other professions named. That any possible trepidation felt by readers in this country is not only useless but probably also groundless, may be gathered from the recent report of Dr. Newsholme, Medical Officer to the Local Government Board. This report shows that while the death-rate from all causes has declined 20 per cent. between 1891-1900 and 1911, there has been a rise in the death-rate among men at ages 45-65. Both in men and women diseases of the heart and blood vessels were the registered causes of about one-third of the total deaths in the age period 55-65. But as there is no evidence that in this mortality dentists led the way, we may properly take comfort of the sort that arises from the assurance that urban professional workers are "all in the same boat."

We would not embark in the risky project of comparing sets of figures compiled by different statisticians and for different countries, but in Dr. Ross' figures relating to dentists in America some pertinent facts may be picked out as a most encouraging offset to his other alarmist conclusions. From 1859 to 1869, the average life was 47.5 per cent.; average time in practice, eighteen years. The next decade 51.7 per cent. years of life; twenty-one years of practice. The next, 56.1 years of life, twenty-eight in practice. Next 57.9 years of life, twenty-eight years in practice. Last decade, from 1899-1909, average age 61.8 years, time in practice thirty-three years. *In fifty years this shows an increase in the length of life 14.3 years, and in of practice an increase of fifteen years.*

*Dentistry as a Department of the Public Health Service.* We have received through reliable sources information that the Board of Aldermen of New York City has recently taken official action placing dental service directly in line with the various other public health activities of the great metropolis. We are informed that—

On recommendation of Dr. E. J. Lederle, Health Commissioner of New York City, the Board of Estimate and Apportionment has appropriated the necessary funds to employ ten (10) dentists by the Department of Health in the division of Child Hygiene, and one (1) dentist in the division of Communicable Diseases. The Board of Aldermen has also passed this and it has been approved by Mayor Gaynor.

In the division of Child Hygiene, nine (9) of the dentists will receive $1200 and one (1) will receive $1500. The one in the division of Communicable Diseases is to be attached to the Otisville Home for Tubercular Diseases, which is under the management of the New York City Department of Health, and will receive $1200.

There was asked for supplies $12,000, and it is probable that this sum has been appropriated, but as the appropriations are made in such a way that some of the items go together in the division of Child Hygiene, the experts who are going over the budget are as yet unable to make a report as to whether this is the exact sum or not.

The appointments have been made from a civil service list, the same as is done in the case of the appointment of physicians for the Department of Health.

This action on the part of the Health authorities of New York City marks another and decided step toward what we believe must ultimately be the disposition of the relationship of dentistry to the public health service, viz, by making dentistry a regular and official factor in the work of municipal and state departments of public health. In its pioneer stages the public health service of dentistry necessarily depended upon volunteer organizations of dental practioners to carry on the work, but each instance of the official recognition of the importance of dental treatment in the public health service becomes a valuable precedent which greatly simplifies the matter of extending such service until all health boards shall have practically included dentistry as a part of their legitimate work.

Philadelphia, we believe, was the first municipality of importance to take the step. The work inauguarated in that city in 1908 has, under the fostering care of the municipal health department, constantly grown, until there are now, besides the Central City Hall clinic, three branch clinics held at various localities in the city, utilizing the services of twelve salaried operators with an increasing demand for the further extension of the free dental service. Wherever the city authorities have stablished a municipal dental service the demand for its extension has naturally followed. Dependence upon voluntary effort for the efficient maintenance of public dental clinics must always constitute a factor of uncertainty. The only guarantee of permanence is the incorporation of dentistry as a part of the organized health service.

Moreover, the official recognition of the value of dental service which is clearly implied in its connection with the organized health work of the municipality and state tends to give to dental service its proper status as a health measure, and to its representatives a professional standing to which, by the very nature of their work, they are justly entitled, but which has previously failed of recognition for the simple reason that its importance has not been either generally understood or recognized by the medical profession as a whole. That such recognition has now been achieved is made evident by the action which we here report upon the part of the New York board of aldermen, and that by the United States government in the passage of the laws creating a commissioned dental service in both army and navy, and by the general demand for skilled dental service growing out of a popular knowledge of the fact that the human mouth is a breeding-ground of pathogenic germs and that it is the principal avenue for the infection of the body.—Dr. Kirk in *Cosmos.*

*Oxygen Poisoning.* Bornstein has been experimenting with animals to determine the conditions permitting life in an atmosphere of hydrogen. He knows of only one case of actual oxygen poisoning in man, and this was on himself while he was studying the subject. The oxygen seemed to affect the lungs exclusively in his experiments; the alveoli were filled with effusion. The experiences reported justify the conclusion that man can stand oxygen at a pressure of two atmospheres for twenty or thirty minutes without harm, which is of interest in connection with general anesthesia.—*Deutsche Med. Wochenschrift* per *Journal of Amer. Med. Association.*

*Iodin in Mouth-washes.* Carles advocates the use of iodin as a mouth-wash, especially in cases of foul breath due to dental caries. One gram of potassium iodid should be added to 20 grams of iodin tincture, and of this mixture from one to three drops should be thrown into a quarter of a glass of warm water. With this solution the mouth should be carefully rinsed. The hotter the water the more drops it will carry. If iodin tincture is used alone, the iodin is separated out by the water, and clinging to the mucous membrane causes a lasting and disagreeable taste. Potassium iodid keeps the iodin in solution, and the taste becomes mild and pleasant. The drug penetrates to all the crevices of the mouth, and acts as a deodorant and an antiseptic. The lotion is to be recommended as a prophylactic to those with sound teeth, and the writer is convinced that by employing it regularly, especially at bedtime, caries can be prevented. The teeth are not permanently stained by the dilution mentioned.—*Gaz. Hebd. des Sciences Med.* per *British Dental Journal.*

*Dentists and General Anesthesia.* It was decided by one of the courts at Paris recently that surgeon dentists who have received a diploma since Nov. 30, 1892, after having taken the necessary studies for the practice of surgery, may use local and general anesthesia in their operations without being obliged to call a physician.—*Journal A. M. A.*

*Digestibility of Bread.* From the point of view of digestibility, fine flour is preferable to course flour; but the microscopic studies of Moeller[1] led him to the conclusion that healthy persons digest the starch of cereals and potatoes almost completely, even when the starchy foods are not in a favorable mechanical condition, as is the case in the bran from cereals, in rice and in sliced potatoes. We maintain that the doctrine of the indigestibility of bread by healthy man is a pernicious and unjustifiable fake.

*Phylacogens Again.* Our practical knowledge of immunity in infectious diseases and of the methods of prophylaxis and cure has advanced greatly during the past ten years. The results of the pro-

[1]Moeller: Ztschr. f. Boil., 1897, xxxv, 291.

phylactic immunization against typhoid and of the treatment of certain cases of furunculosis are familiar examples of what may be accomplished by active immunization with preparations of bacteria commonly known as vaccines. Definite and favorable results have been obtained by the use of vaccines prepared from the organisms concerned in various other acute and chronic infections. The results from the use of vaccines have appealed to physicians generally, and the use of vaccines in many infections has become more and more widespread. Vaccines for these inoculations are supplied by the biologic departments of drug houses, and the preparation and sale of these products have come to occupy a prominent and in all probability a remunerative department, if we may judge by the space devoted to their advertising.

The thinking physician knows, however, that the treatment of infection with vaccines requires careful attention and thought, together with a study of the clinical and pathologic conditions of the individual case, and that unless this requirement is met, more failures than successes follow. We do not enter here into a discussion of the relative merits of stock and autogenous vaccines, or into the question as to what combination of mixed vaccines may be desirable. We assume that the average physician is doing his best with products supplied by the laboratories and that he has not the facilities for the preparation of autogenous vaccines. Under these conditions the problem of obtaining results is difficult enough, and the physician does not have to go far before he finds that even with the most painstaking work he is often unable to accomplish the cure of the infection in question, the consequence being that at present vaccines are discredited by many physicians.

And now the physician is asked practically to disregard the little knowledge he already has of the mechanism of infection and inject into his patients a mixture of toxic bacterial derivatives, called Phylacogens, and see what will happen. Something usually does happen, and the patient has good reason to remember the experience of the chill and violent constitutional symptoms that follow the injection. It hardly seems possible that physicians of experience ever would countenance the injection of such toxic substances into patients already overwhelmed with the poisons of infection, such as that by the streptococcus in erysipelas or the pneumococcus in pneumonia.

To complete the list of Phylacogens so that the busy practitioner need not waste his time in thought, provided he is unable to determine on clinical grounds the nature of the illness in his patient, he is provided with Mixed Infection Phylacogen, a preparation which has the merit of covering the ground, but only so far as name is concerned. We are inclined to look with pity on the poly-pharmacy of earlier centuries in medicine, and yet in this day of research into the specific causes of infection and of specific treatment we are asked to introduce into patients suffering from a known infection a mixture of many toxic substances, the action of no one of which is understood. This is an utterly irrational procedure, without any semblance of adequate experiemtal and theoretical basis, and in the end it will work great harm to the cause of legitimate and rational vaccine therapy, to say nothing of the harm inflicted on the innocent patient.—*Journal A. M. A.*

*Prophylaxis of Cancer.* Theilhaber's views in regard to the necessity for warding off recurrence of cancer and for precention of cancer in general were summarized in these columns Oct. 19, 1912, p. 1499. He here reiterates that cancer never develops in perfectly normal tissues, and that by keeping the vitality of the tissues up to par there will be no cancer. Any apparently trifling injury of the breast should be treated with hyperemia, massage or diathermia, as most convenient, or all combined. In this way he is convinced that many cancers will be warded off. The same principle should be applied to scar tissue anywhere, especially for persons who have already had a cancer removed. The family might be trained to apply the necessary prophylactic measures. More than 75 per cent. of recurring cancers develop in the cicatrix. It seems that Theilhaber had been advocating the application of heat in prophylaxis of cancer long before Bier published his methods of hyperemia treatment.—*Journal A. M. A.*

*Occupation of the Parents as Factor in Development of Child at Birth.* Goldfeld gives tables to show the average weight and size of the children of teachers, hotelkeepers, merchants, butchers, carpenters, bakers, masons, mechanics, factory workers and printers, classifying the children as they were the first, second, third or later children. The research was undertaken at Wurzburg and showed several interesting general rules; for instance that the later children average 116.5 gm. more in weight than the first child. The weight of the child at birth seems to increase with the age of the mother, and the boys weigh more than the girls. The physical development of the child seems on a par with the financial standing of the parents. It is highest in the families of teachers and lowest among the day laborers. It is striking that the standard is so high in factory workers' families, and Goldfeld ascribes this to the national insurance, the societies writing industrial insurance having inaugurated measures to conserve the health of women during child-bearing. The tables show anew the familiar fact that those who are less financially fitted for it are the ones who procreate with the least scruples.—*Journal A. M. A.*

*Deforming Arthritis.* Jacobsohn illustrates his article with twenty-eight Roentgen pictures, aiming to establish that hypertrophic arthritis is a well defined morbid entity. It should be distinguished from other joint affections, especially those with a tendency to degenertive atrophy. He objects to the name deforming arthritis, as the characteristic feature of the affection is the hypertrophy, while this symptom of hypertrophy is not an essential feature in any other joint disease. There is little chance for restitution. Treatment should be based on the axiom, "Avoid rest, keep the affected joint well exercised." Repose may alleviate or cure the pain, but under rest the muscles are liable to atrophy. It should be the physicians' task to prevent the development of atrophy; only exceptionally should long rest in bed or immobilization be allowed. Too often under rest and sparing the joint the muscles become atrophied, interfering with the gait and causing weakness and edema, impelling the patient to spare the joint more and more until weeks and months may be required to undo the effects if this is ever possible. The physician should insist on exercise of the joint, informing the patients that the trouble is of a chronic character, that it may flare up now and then, and the joint will never be entirely normal again, but that the affection is harmless and work should not be given up on its account as movements of the joint in manual labor guarantee a better out come than sparing the limb. When there is severe pain in walking and standing, he applies massage and medico-mechanical measures, supplemented by hyperemia treatment (hot air or suction apparatus) and faradization. When the hip joint is involved an orhtopedic apparatus may be come necessary but operative intervention is only exceptionally to be considered—only when loose bodies cause pain or become lodged in the joint. He describes the clinical picture and roentgen findings in the various articulations in turn.—*Journal A. M. A.*

*Novocain.* We have used novocain in the dental clinic at the University of Pennsylvania quite extensively, and it has come to us through two sources; first, the insistence of Dr. Prinz upon its use as an anesthetic, and the large number of students who come to us from Europe and have been quite accustomed to use novocain in the hospitals in Europe, have insisted upon using it in our clinic in preference to cocain. Our experience with it bears out the reports of Dr. Riethmuller on the European experience with it. The very dimished toxicity of novocain as compared with that of cocain is probably true—at least we have found it to be true in our experience; the features, too, of no after-effects and the facility with which it can be sterilized by heat—all are definitely in its favor. While it is not as rapid in its anesthetic effect as cocain, if given a little time it will produce an anesthesia quite as profound and lasting or a little more so than cocain. We have come to regard it as a very great step in advance in the means for producing local anesthesia.

The question which Dr. Gaskill raised as to whether there is danger of necrosis is an important one. I do not apprehend on general principles that there would be any danger of producing necrosis of bony tissue with sloughing of the soft tissues by injections of novocain, providing the operation is done aseptically. That is to say, the toxicity of the drug is relatively so slight that I do not apprehend any serious necrotic effect from the poisonous character of the drug itself, and by using isotomic solutions any danger from the difference in specific gravity is eliminated. Statistics of operations thus far done, which cover now a record of half a million cases, as Dr. Riethmuller has cited, seem to indicate that we have in novocain a safe and reliable means of producing local anesthesia, and one which very certainly eliminates a number of the disavdantages of local anesthesia with cocain, especially the very great uncertainty as to what is the minimum safe physiological dose of cocain. We know the idiosyncratic relation of people to that drug, and that many will be almost alarmingly affected by a fair average dose. I do not mean psychologically alone, but that a definite physiological disturbance is produced in the circulation of such patients by even a minute dose of cocain, and that tricky quality does not seem to attach itself to novocain.—Dr. E. C. Kirk in *Cosmos*.

*Get Busy.* The season of dental meetings is coming on, and it behooves every dentist to look the situation over very carefully. A new order of things is on the way, and the old order is all but gone. For the first time in the history of dentistry a definite system of co-related societies is ready for the banding together of dentists in one substantial organization, which will have power and influence such as never existed before. This organization makes possible so many good things in the way of further education and such possibilities in securing needed legislation that the best informed have hardly grasped its full meaning.

No dentist can possibly afford to remain without society affiliations in the face of these new conditions. There may have been excuses for not taking part in dental society work in the past, but there can be none now. A new era of progress is bound to come from now on, and a man must be in touch with the whole matter all the time to get the good out of it. The man who just sits back and looks on is going to get left in the procession. There never was a time like right now for getting in at the beginning and extracting the good out of everything.

Those who take part now and help shape up policies and plans may help shape them up right along the lines most wanted. No need to let some other bunch of fellows fix things and not fix them to your liking. You can now make your needs and your wishes known and felt quite as much as mine or some other fellow's. If you have in mind some plan you would like to see adopted for the betterment of yourself and your profession, you will no longer be a single individual working alone. You can associate with others of your kind, and anything of interest or merit can be brought to the front and attention compelled.

Suppose you are in favor of dental inspection in the public schools, or dental attention to the inmates of our public institutions, or any of the dozens of good things that have been proposed. You can now work effectively to accomplish something. By getting your friends and your society interested you can get other men and other societies interested. There are always plenty of champions for any good cause, and when these have some effective way of co-operating, things can be done that individuals could never accomplish.

Every dentist should take part in society work under the new conditions, not only to further his own ideas and wishes, but to help others further theirs. If some other fellow believes in the same things as you do, and works in his society to accomplish something, he needs you to work in your society for the same ends quite as much as you need him. You have a chance to be helper as well as helpee.

Every dentist who is worth two cents a dozen has some good idea for the benefit of others. In times past he may have felt the uselessness of trying to push good ideas under the old, disjointed society methods, but now it's different. That is, it will be different if he jumps in and makes himself a society man. And there's just the point—he *must* be a society man, a dental society man, we mean—if he's going to get into the new ways. If things are to be accomplished through organization the accomplishers must "belong". Nothing can be done by divided forces, some on the outside and some on the inside. The bunch must be together to carry the ward.

The new plans for the National Dental Association provide that membership may only be held through membership in a component society, and reversing the proposition, being a member of the component (local) society carries with it membership in the higher society. While all the details have not been worked out, this is the essence of the plan, and in a very few years dentistry will be getting the benefit of this compact and interdigitating system. While it will benefit all dentists in a way, it will benefit its members for the most, and in many details will benefit *only* the members. This is as it should be. Why should the Jebusites and Philistines have the same rewards as the faithful, who do all the work? Verily, they won't. The Philistines may seemingly flourish without the gates of the city for a time, but before long they will be as grass before the reaper. That is, they won't be in it with the dental society members.

Elsewhere we give, under dental society announcements, the officers of a number of state and local societies, who will be glad to arrange for membership with any reputable practitioner. If there is a single reader of this journal who is yet without membership in his local dental society, he is doing an injustice to himself, his patients, his profession, his family and his friends. Get busy.—Dr. Allen in *Western Journal.*

*Oral Hygiene in Cincinnati Schools.* The recognition which the oral hygiene movement has found in the medical, educational, and administrative camps is signally attested by the liberal space allotted by this medical journal to the oral hygiene symposium arranged by the Cincinnati Dental Society on April 26, 1912. The first author, chairman of the meeting, defines the oral hygiene movement as consisting of three main divisions. Educational work such as public lectures, tooth-brush drills, etc.; dental inspections, and the free clinic, which, in his opinion, should be taken over by the municipality, this policy being cheaper in the end than the child's failure of promotion in the public school.

Dr. Geier, director of charities and corrections, expresses himself as fully appreciating the helpfulness and need of dental work in the house of refuge, the first purpose of which is to restore the children to a normal physical condition.

Dr. Dyer, superintendent of public schools, is of the belief that the oral hygiene movement in schools has been the most important one for the profession as well as the children, that has transpired in the history of the dental profession. He reports that, in 1909, nine hundred and twenty children in one of the schools under him were examined, of whom less than ten had had dental attention. An examination in the same school in 1910 showed that 25 per cent., viz, two hundred and fifty children, had received dental treatment, which speaks loudly for the awakening of the people.

Mr. Hauer, school principal, testifies to the effect that the dental work done in his school has proved to be mentally, morally, and physically beneficial to the pupils, whose parents in a four-fifths majority cannot afford to pay for dental work.

Dr. Porter cites the appalling statistics revealed by dental examinations of school children, and comments upon the dispatch with which the dental profession has overcome the initial skepticism of the medical profession toward this movement. He recommends free dental treatment ofchildren even before they have reached school age, and considers that at present there are no philantropic movements that offer greater opportunities than this plan of looking after the physically deficient children who otherwise would be neglected. It is infinitely better to give these children a fair chance to grow up into useful, independent citizens than to organize charities for them after they have reached a state of partial or entire dependence, as a result of ailments that could have been corrected in childhood. We hear much of the awful danger of race suicide, but instead of weeping over hypothecical children, we will do much better to devote ourselves to the welfare of the actual flesh-and-blood children with which we are surrounded.

Dr. Iglauer, president of the Cincinnati Anti-Tuberculosis League, complains that the dental and anti-tuberculosis movements are greatly handicapped, because both are directed against diseases so widespread throughout the world and so insidious in their onset. The diseases which both movements seek to overcome have been with us so long, and society has become so accustomed to their presence, that by some they are regarded as almost inevitable. Familiarity with disease often breeds contempt for the same, so that preventive dentistry and medicine have to encounter the ignorance, superstition, and inertia of a public unmindful of its own best interests. Propaganda must therefore be made through lectures, the press, public clinics, and above all through the public schools, to enlighten the young and prospective adult generation of citizens and voters, because the solution and control of all movements for the public health must finally rest with the state. The formation of a national bureau of health, so long delayed but now in prospect of consummation, is a great step in the right direction, but the state and the city also have an important part to perform in this great work toward final victory over preventable diseases.

M. Edith Campbell, of the Cincinnati board of education, states that one of the most difficult tasks of her office is to convince the wage-earning girl that her health is her capital. Since all methods of reform, and attempts at class legislation toward producing the efficient, well-developed human being have failed so far, we are forced to the old wearying way—the way of the individual. The individual must be taught that only through the perfect care, conservation, and development of his physical and mental resources can he compel life to disclose its secret richness to him, and can he conquer its exigencies and rise above its sordid struggle. In such a training of the individual the dental work in public schools will be a most potent force.

Dr. Landis, health officer of Cincinnati, emphasizes the untoward influence of unhealthy oral conditions on the entire organism, and points out that defective teeth and unclean mouths mean a great financial loss to a community, because it requires children thus affected at least six months longer to complete the eight common school grades than it does those without defective teeth. The expense incurred by public dental school clinics, therefore, is a real economy and the oral hygiene movement will bring about more perfect physical development, greater freedom from preventable disease, a shorter average period in school, and a wider dissemination of information concerning hygiene.—*Cosmos.*

